# Vertebral fracture during one repetition maximum testing in a breast cancer survivor: A case report

**DOI:** 10.1097/MD.0000000000025705

**Published:** 2021-05-21

**Authors:** Friederike Rosenberger, Justine Schneider, Kathrin Schlueter, Jean-Luc Paratte, Joachim Wiskemann

**Affiliations:** a Working Group Exercise Oncology, Department of Medical Oncology, National Center for Tumor Diseases (NCT) Heidelberg, Heidelberg University Hospital; b Division of Health Sciences, German University of Applied Sciences for Prevention and Health Management, Saarbrucken; c Institute of Sports and Sport Science, Heidelberg University, Germany.

**Keywords:** adverse event, case report, resistance training, safety

## Abstract

**Rationale:**

One repetition maximum (1-RM) testing is a standard strength assessment procedure in clinical exercise intervention trials. Because no adverse events (AEs) are published, expert panels usually consider it safe for patient populations. However, we here report a vertebral fracture during 1-RM testing.

**Patient concerns:**

A 69-year-old breast cancer survivor (body-mass-index 31.6 kg/m^2^), 3 months after primary therapy, underwent 1-RM testing within an exercise intervention trial. At the leg press, she experienced pain accompanied by a soft crackling.

**Diagnosis:**

Imaging revealed a partially unstable cover plate compression fracture of the fourth lumbar vertebra (L4) with a vertical fracture line to the base plate, an extended bone marrow edema and a relative stenosis of the spinal canal.

**Interventions:**

It was treated with an orthosis and vitamin D supplementation. Another imaging to exclude bone metastases revealed previously unknown osteoporosis.

**Outcomes:**

The patient was symptom-free 6.5 weeks after the event but did not return to exercise.

**Conclusion:**

This case challenges safety of 1-RM testing in elderly clinical populations.

**Lessons:**

Pre-exercise osteoporosis risk assessment might help reducing fracture risk. However, changing the standard procedure from 1-RM to multiple repetition maximum (x-RM) testing in studies with elderly or clinical populations would be the safest solution.

## 1 Introduction

Resistance training has gained more and more importance for health promotion in the general population as well as in different clinical populations over the past decades.^[1–5]^ In exercise intervention studies, one repetition maximum (1-RM) testing represents a standard procedure for dynamic strength assessment using training machines.^[6,7]^ The 1-RM is defined as “the greatest resistance that can be moved through the full range of motion in a controlled manner with good posture” and the test is considered valid and reliable (ICC > 0.99) also in untrained populations.^[6,7]^

With regard to safety of 1-RM testing in untrained and clinical populations, a study from the 1990 s in 74 cardiac rehabilitation patients revealed no adverse event (AE).^[8]^ Another study from that period in 83 participants of a geriatric fitness program reported 2 AEs during 1-RM testing, a rib fracture and a back injury, in the subgroup of individuals with no prior resistance training experience.^[9]^ It is concluded that proper preparation provided 1-RM testing can be a safe assessment tool in the elderly.^[9]^

Today, the number of exercise intervention studies in cancer survivors increases rapidly and 1-RM testing is widely used for strength assessment.^[10–27]^ While there is no literature available especially on the safety of the 1-RM testing in cancer survivors, reviews on the safety of exercise intervention trials in general exist: Speck et al^[28]^ reviewed 82 studies of which 44% reported the presence or absence of AEs. One of those studies found 5 exercise-related AEs (increased blood pressure, hip pain, pulled hamstring, fall, calf pain). Similarly, Singh et al^[29]^ reviewed 61 studies with special focus on advanced cancer of which 41% reported the absence or presence of AEs. In total, 6 exercise-related grade 3 AEs were found (discomfort, dizziness and dyspnea, syncope, mild chest pain, foot pain, unspecified physical accident) and no grade 4/5 AE. Altogether, AEs were not more common during resistance training compared to other training modalities. These findings do not point towards frequent injuries during strength assessment in cancer survivors. Consequently, the latest exercise guidelines for cancer survivors by the International Multidisciplinary Roundtable^[3]^ state “the evidence-based literature indicates 1-RM testing is safe among survivors of breast and prostate cancer without bony metastases”. However, since the majority of studies in the mentioned reviews did not report on the presence or absence of safety, underreporting of AEs cannot be excluded.

Against this the backdrop of increasing numbers of studies in clinical populations, frequent use of 1-RM testing and potential underreporting of safety issues, it appears important to report and discuss a serious adverse event that occurred in a study in our laboratory during 1-RM testing at the leg press in a breast cancer survivor. The patient gave written informed consent to publish her individual clinical data here.

## 2 Case report

### 2.1 Setting

The serious adverse event occurred within a randomized controlled four-arm exercise intervention trial in breast and prostate cancer survivors after the end of primary therapy (TOP study, clinicaltrials.gov NCT02883699). The trial investigated the effects of different training regimens on performance changes. Two of the arms were resistance training arms with 20 participants planned in each arm. The trial was approved by the Medical Faculty Heidelberg Ethics Committee (S-347/2016) and followed the declaration of Helsinki.

All participating breast cancer survivors fulfilled the following inclusion criteria:

1. diagnosed with non-metastatic breast cancer,
2. 6 to 52 weeks after end of primary therapy,
3. 18 to 75 years of age,
4. no regular vigorous endurance or resistance training (>1 session/wk) since diagnosis or within the last 6 months.

Exclusion criteria were:

1. additional other cancer or
2. any comorbidities that preclude participation in exercise testing or training (e.g., acute infectious diseases, severe cardiac, respiratory, renal or neurological diseases).

### 2.2 Patient information

One participant was a 69-year-old women (height: 170 cm, weight 91.4 kg, body-mass-index 31.6 kg/m^2^) who had been diagnosed with breast cancer (cT2 cN+ G3 ER+ PgR0%) 13 months ago. She had underwent neoadjuvant chemotherapy, segment resection of the right breast with lymph node dissection, and radiotherapy until 3 months ago. She did not receive anti-cancer hormone treatment (i.e., tamoxifen or aromatase inhibitors). Known co-morbidities were

1. intervertebral disc protrusions at third/fourth lumbar vertebra (L3/L4), L4/fifth lumbar vertebra (L5) and L5/ first sacral vertebra (S1), diagnosed 5 years ago and currently symptom-free,
2. osteochondrosis at the eleventh thoracic vertebra, currently symptom-free,
3. hypothyroidism,
4. hepatic cysts,
5. glaucoma,
6. chemotherapy-induced peripheral neuropathy, and
7. sarcoidosis as side effect of the anti-cancer treatment.

Current medication was thyroxin and eye drops. The participant was randomized to one of the 2 resistance training arms.

### 2.3 Adverse event

Baseline strength assessment included isometric and isokinetic testing on a stationary dynamometer (IsoMed 2000 B-Series version, D&R Ferstl, Hemau, Germany) as well as 1-RM testing at 6 training machines in fixed order: leg flexors, rowing machine, leg extensors, lat pulldown, leg press, and shoulder press. The participant performed isometric and isokinetic strength testing as well as 2 training sessions at the machines for familiarization without problems.

In the third training session, 1-RM testing was performed by an experienced certified exercise therapist. The exercise therapist explained the test, checked posture and supervised movement during a machine specific warm-up of 10 repetitions with low resistance. Then, the first testing resistance was selected so that the patient was likely able to lift it once with appropriate technique through the complete range of movement. If this attempt was successful, resistance was increased until the 1-RM was reached. The total number of attempts should not exceed 5 and 2 minutes resting periods were applied between attempts.

At the first 4 machines, 1-RM testing was performed without problems. At the fifth machine, the leg press (supine position), the participant performed 4 successful attempts with 77, 85, 93, and 101 kg. The fifth attempt with 109 kg failed and when pressing, the participant took a slightly kyphotic position of the lumbar spine (against the exercise therapist's instruction and too fast to intervene) and immediately felt pain in the lower back accompanied by a soft crackling. The pain was not too severe and the testing session was completed at the sixth machine without further problems. When sending the participant home, the therapist advised her to visit an orthopedist if pain persists. The exercise therapist also informed the study team about the event. They called the participant and advised her again to visit an orthopedist if pain persists.

### 2.4 Diagnostic assessment and clinical findings

The participant first believed to suffer from lumbago without structural damage and took ibuprofen 600 mg according to demand for 3 days. When pain persisted for 2½ weeks, she visited an orthopedist. The anamnesis and physical examination revealed decreasing pain since the event and no neurological findings. A computer tomography (CT) and a magnetic resonance imaging (MRI) revealed a cover plate compression fracture (partially unstable) of L4 with a vertical fracture line to the base plate, an extended bone marrow edema and a relative stenosis of the spinal canal. The following further findings were previously known: an intervertebral disc protrusion L3/L4 with high degree constriction of the spinal canal and low liquor signal, an intervertebral disc protrusion L4/L5 with a relative constriction of the spinal canal as well as a calcification of the disc space L5/S1. To exclude an unknown osseous metastasis, a further MRI with bone puncture was performed. No tumor cells were found. However, a previously unknown osteoporosis was reported but not closer classified.

### 2.5 Therapeutic intervention

The fracture was treated with an orthosis (T-FLEX TL basic) worn during the day for 4 weeks and vitamin D supplementation. The participant reported strict adherence and high tolerability of this intervention.

### 2.6 Follow-up and outcomes

Four weeks later (or 6.5 weeks after the event), the participant was symptom-free and felt well. No unexpected or further adverse event occurred until 8 weeks after the reported event, but the participant did not continue study participation or want to return to machine-based resistance training. She was classified as a drop-out due to a serious adverse event related to exercise testing. The study design was not modified after this event (i.e., 1-RM testing was continued) and no further fractures occurred in the remaining 39 participants of the resistance training arms in this study.

## 3 Discussion

This case report of a vertebral fracture during 1-RM testing at the leg press in a 69-year-old breast cancer survivor demonstrates that 1-RM testing might not be as safe as supposed and recently stated in the International Multidisciplinary Roundtable's exercise guidelines for cancer survivors.^[3]^ Because AEs were potentially underreported in previous studies,^[28,29]^ this AE appears worth discussion to prevent similar cases in future.

First, it shall be addressed whether and how the fracture during 1-RM testing could have been avoided. Here, the most relevant point is the previously unknown osteoporosis. The International Multidisciplinary Roundtable's exercise guidelines for cancer survivors states that “among patients with bony metastases or known or suspected osteoporosis routine assessments of muscle strength and/or endurance involving musculature that attaches to and/or acts on a skeletal site that contains bone lesions should be avoided”.^[3]^ In this case, osteoporosis was neither known nor suspected by the study team. The participant was a relatively old postmenopausal woman but she did not have a history of fractures and did not receive anti-cancer hormone therapy or other medication known to increase the risk of osteoporosis.

Pre-exercise evaluation tools to “know” or “suspect” osteoporosis were not applied in this case, but might have avoided the AE. To diagnose osteoporosis, bone mineral density measurement using dual-energy X-ray absorptiometry (DXA) is the gold standard.^[30,31]^ However, DXA is expensive and associated with a small radiation exposure. For the latter reason, ethic committees in several countries apply a restrictive policy concerning its scientific use. There are also scores available to assess the risk of fragility fractures. These scores include risk factors like age, sex, body-mass-index, family history of hip fractures, glucocorticoid treatment, smoking, alcohol intake, height loss, or thoracic kyphosis.^[31]^ Out of these scores, the clinical fracture risk assessment (FRAX) is recommended by American and European expert panels.^[30,31]^ It can be used with or without DXA data. However, when retrospectively applying FRAX without DXA in the present patient, the risk of major osteoporotic fracture within the next 10 years is only 6.5%. Altogether, DXA would probably have prevented the AE in this case but FRAX alone would have not. This does not mean that FRAX is not helpful per se and it might be applied in future studies given its low costs, time requirements and the absence of ethical concerns. But the present case suggests that for safe 1-RM testing DXA-based exclusion of osteoporosis might be required in postmenopausal women from a certain age on.

Beyond that, a safer alternative to 1-RM testing shall be discussed. For dynamic strength assessment using training machines, the multiple repetition maximum (x-RM) test is suggested as another option by the American College of Sports Medicine.^[7]^ It reflects the greatest resistance that can be moved several times, for example, 4, 8, 10, or 12 times. Osteoporosis guidelines might help finding a safe x-RM test for populations with potentially fragile bones. The Delphi consensus on exercise recommendations for adults with osteoporosis^[32]^ states that individuals with osteoporosis should train at the 8- to 12-RM (with the exception of trunk flexors or extensors). This indicates that 8- to 12-RM testing should be safe when making sure that the last failed attempt does not exceed the 8-RM too much (i.e., using small increments in weight). In the present case, the AE would not have occurred in an 8- to 12-RM test because the participant safely performed attempts up to 101 kg which was so close to the 1-RM that 8 or more repetitions would not have been possible. The authors of this case report therefore recommend a change in the standard procedure from 1-RM to x-RM testing (i.e., 8-, 10-, or 12-RM testing) in studies with elderly or clinical populations.

A strength of this case report is that 1-RM testing was performed by an experienced exercise therapist under standardized conditions following textbook procedures. The AE is therefore not attributable to incorrect procedures. Furthermore, the breast cancer survivor was screened for study participation using standard inclusion and exclusion criteria. She is therefore representative for participants in exercise oncology studies and, because there was no cancer-specific cause or risk factor for the fracture, even for elderly clinical study populations in general. However, a potential limitation of this case report is that more detailed clinical data on the participant's osteoporosis status are lacking. Even if this does not have any consequences on the adverse event because it was diagnosed thereafter, the discussion is based on it and information on the severity of the osteoporosis would have helped, for example, to estimate the likelihood of such events.

## 4 Conclusion

This case of a 69-year old breast cancer survivor who experienced a vertebral fracture during 1-RM testing within an exercise intervention trial questions the safety of 1-RM testing in elderly clinical populations. The fracture was probably promoted by osteoporosis, which was diagnosed after the event. This highlights the need of special pre-exercise screening. A combination of DXA in defined subpopulations like elderly women and the general use of a fracture risk assessment score like FRAX might enhance safety of 1-RM testing. However, an even more secure solution would be a change in the standard strength assessment procedure from 1-RM to x-RM (i.e., 8-, 10- or 12-RM) testing in studies with elderly or clinical populations.

## Author contributions

**Conceptualization:** Friederike Rosenberger, Joachim Wiskemann.

**Data curation:** Friederike Rosenberger, Justine Schneider, Kathrin Schlueter, Jean-Luc Paratte.

**Formal analysis:** Friederike Rosenberger, Jean-Luc Paratte.

**Funding acquisition:** Friederike Rosenberger, Joachim Wiskemann.

**Investigation:** Friederike Rosenberger, Justine Schneider, Kathrin Schlueter, Joachim Wiskemann.

**Methodology:** Friederike Rosenberger, Jean-Luc Paratte, Joachim Wiskemann.

**Project administration:** Justine Schneider, Kathrin Schlueter.

**Supervision:** Friederike Rosenberger, Joachim Wiskemann.

**Writing – original draft:** Friederike Rosenberger.

**Writing – review & editing:** Friederike Rosenberger, Justine Schneider, Kathrin Schlueter, Jean-Luc Paratte, Joachim Wiskemann.
